# Addressing COVID-19 Barriers to Clinical Trial Enrollment and Implementation in the PHARM-DC Study

**DOI:** 10.1093/geroni/igab046.393

**Published:** 2021-12-17

**Authors:** Joshua Pevnick, Michelle Keller, Korey Kennelty, Michelle Ko, Logan Murry, An Nguyen, Andrew Henreid, Jeffrey Schnipper

**Affiliations:** 1 Cedars-Sinai Medical Center, Los Angeles, California, United States; 2 Cedars Sinai, Cedars Sinai, California, United States; 3 University of Iowa, Iowa City, Iowa, United States; 4 Brigham and Women's Hospital, Boston, Massachusetts, United States; 5 Cedars-sinai Medical Center, Los Angeles, California, United States

## Abstract

Recent hospitalization puts older adults at higher risk of experiencing adverse drug events (ADEs) that are a common cause of hospital readmission. Yet, most ADEs are preventable. The PHARMacist Discharge Care (PHARM-DC) study is a multi-site randomized controlled trial that seeks to evaluate the effect of pharmacist-led peri- and post-discharge interventions on 30-day hospital readmissions among older adults taking ≥10 medications or ≥3 high-risk medications. The PHARM-DC intervention includes pharmacist-led patient counseling, medication reconciliation at discharge, and a follow-up phone call post-discharge. We will highlight study protocol adaptations undertaken during the COVID-19 pandemic to address challenges to enrollment and to minimize risk of COVID-19 exposure for study participants and research personnel. Additionally, we will share insights from focus groups and semi-structured interviews with pharmacist interventionists and pharmacy leaders on barriers and facilitators to implementation due to the pandemic and strategies for future clinical trials to overcome barriers.

